# Supplementary material for: Sustained-input switches for transcription factors and microRNAs are central building blocks of eukaryotic gene circuits
Source: Genome Biol. 2013 Aug 23;14(8):R85. doi: 10.1186/gb-2013-14-8-r85 (PMC4054853; doi:10.1186/gb-2013-14-8-r85)
Supplement: Additional file 5 — HTML Browsable Motif Output. Zipped folder containing all WaRSwap and FANMOD motif output, viewable in a web browser. [file gb-2013-14-8-r85-S5.ZIP › HTML_browsable_motif_output/WaRSwap_all_tair9/sigs_WSWRSLDraw-2000_all.pvals.heatmaps.html/motif_id_38_001101001_tftype_all_upstream_-1000_0.html]

```
BG_MODEL = Rnew2
MOTIF_ID = 38_001101001
TF_TYPE = all
UPSTREAM = -1000_0


PVals
FN_0.2	FN_0.4	FN_0.6	FN_0.8
dg_60.genes	0.995	0.468	0	0.684
dg_70.genes	0.998	0.433	0	0.689
dg_80.genes	0.996	0.455	0	0.683

ZScores
FN_0.2	FN_0.4	FN_0.6	FN_0.8
dg_60.genes	-2.595	0.096	3.896	-0.519
dg_70.genes	-2.64	0.153	3.864	-0.519
dg_80.genes	-2.585	0.13	3.898	-0.548

StDevs
FN_0.2	FN_0.4	FN_0.6	FN_0.8
dg_60.genes	6529.358	961.376	194.477	28.918
dg_70.genes	6517.992	945.152	196.666	29.434
dg_80.genes	6509.562	942.355	194.243	29.357
```
